# Clinical genetics of melanoma

**DOI:** 10.1186/1897-4287-10-S1-A6

**Published:** 2012-01-12

**Authors:** T Dębniak

**Affiliations:** 1 International Hereditary Cancer Center, Department of Genetics and Pathology, Pomeranian Medical University, Szczecin, Poland

## 

Malignant melanoma (MM) represents one of the most aggressive neoplasms and its frequency is increasing rapidly. Increased melanoma risk among relatives of MM patients and familial aggregations of this malignancy point at genetic predisposition as an important factor of MM pathogenesis. The genetic basis of MM is complex and appears to involve multiple genes. CDKN2A is regarded as the major MM susceptibility gene. In the Polish population common CDKN2A variant (A148T) increases significantly melanoma risk regardless of the cancer family history. Mutations of other high risk genes, ARF and CDK4 are extremely rare and thus clinically insignificant. 

The higher rates of CDKN2A/p16 mutations can be detected only in rare cases characterized by: 

1) occurrence of three or more primary melanomas,

2) patients with three or more melanomas in aggregate among first or second degree relatives,

3) families with the presence of three or more cases of melanoma and/or pancreatic cancer on the same side of the family. It is thus necessary to perform association studies focused on identifying genetic markers that could be used in identifying patients with a high risk of MM. List of other genes that carry mutations, which are believed to be associated with moderate MM risk include XPD, MC1R, BRCA2. Newest genome-wide association study identified three loci associated with melanoma risk: 16q24, 11q14-q21, 9p21.

The management with individuals being at increased MM risk involves clinical screening according to carefully planned surveillance schedule and early treatment of MM tumour. The appropriate management may reduce morbidity and mortality. Genetic testing and clinical evaluation should be performed, and family history should be obtained in all patients affected with MM, also in those with apparently sporadic tumors.

